# Ethical considerations for informed consent in infertility research: The use of electronic health records

**DOI:** 10.12715/ame.2015.2.7

**Published:** 2015-12-21

**Authors:** Kristen J. Wells, Janna R. Gordon, H. Irene Su, Shayne Plosker, Gwendolyn P. Quinn

**Affiliations:** 1 Department of Psychology, San Diego State University, San Diego, CA, USA; 2 Moores Cancer Center, University of California, San Diego, La Jolla, CA, USA; 3 San Diego State University/University of California, San Diego Joint Doctoral Program in Clinical Psychology, San Diego, CA, USA; 4 Division of Reproductive Endocrinology and Infertility, University of California, San Diego, La Jolla, CA, USA; 5 Department of Obstetrics and Gynecology, Morsani College of Medicine, University of South Florida, Tampa, FL, USA; 6 Health Outcomes and Behavior Program, Moffitt Cancer Center, Tampa, FL, USA; 7 Department of Oncologic Sciences, Morsani College of Medicine, University of South Florida, Tampa, FL, USA

## Abstract

The growing use of electronic health records (EHRs) in healthcare provides rich opportunities for biomedical research. Using EHRs, massive quantities of patient data can be extracted for research without the need to recruit patients, schedule study visits, or rely on self-reporting. However, this innovation poses significant concerns about patient privacy and confidentiality of data. Patients receiving infertility treatment may be particularly vulnerable to data breaches, as their EHRs often include sensitive health information about themselves, their partner, and their offspring. Helping patients with infertility to make informed decisions about sharing data is crucial, yet little is known about best practices for obtaining informed consent to use EHR data for research. This commentary reviews possible options for obtaining informed consent for EHR use among patients seeking fertility services. In addition, this commentary summarizes the limited research available on patient preferences for informed consent practices.

## Commentary

Approximately 7 million couples in the United States are infertile [1]. Infertility is defined as having the desire for a biological child and attempting pregnancy through sexual intercourse without success, for at least one year if the woman is under 35 years of age, or six months if the woman is 35 years of age or older [2]. Treatment options for infertility range from simple consultations to invasive medical interventions [3]. Basic consultations provide advice on timing of sexual intercourse and changes in lifestyle to enhance fertility. However, if these procedures are unsuccessful and the couple still wishes to proceed, a battery of tests and examinations may be conducted with both partners. Standard diagnostic evaluations range from blood tests and semen analysis to more invasive tests of reproductive organs. Depending on the cause of infertility, treatments can include administering medications to induce ovulation, intrauterine inseminations, surgeries to correct anatomic abnormalities, and in vitro fertilization [3–5].

Patients who seek fertility counselling are required to disclose a great deal of potentially sensitive personal and family health information, which may be stored in electronic health records (EHRs). EHRs are real-time patient-centered records that can contain patient health information [6], and – in the case of patients seeking fertility services – may include sexual health history, prior pregnancies and elective abortions, chronic or acute conditions, family health history, and information about a partner’s health. Additional tests may be performed, such as genetic analysis, sexually transmitted infection screens, pelvic imaging, and blood draws, as well as recommendations for oocyte donation and gestational surrogacy. The outcomes of fertility treatments documented in the EHR can include the results of genetic testing of embryos.

EHRs have the potential to “revolutionize the health care research enterprise” by creating large data banks of information that can be used for biomedical research [7, 8]. Because data are entered and become available in almost real time, patient data of interest can be identified and extracted without the need to recruit and schedule research study visits, and without relying on patient recall in surveys. Widespread use of EHRs may enable the development of a Learning Healthcare System, spearheaded by the Institute of Medicine [9]. In practice, a Learning Healthcare System requires the massive amounts of data contained in EHRs nationwide (e.g., health centers, medical practices, and health agencies) to be extracted and moved to investigative “centers” where they are aggregated into large data sets that can be routinely analyzed to answer research questions, such as monitoring adverse effects of a new drug [7]. The use of EHR data in biomedical research provides rich opportunities for expansion of biomedical knowledge; however, the possibility of mishandling data and privacy breaches [10] are significant concerns [11].

Little is known about best practices for helping patients make informed decisions about whether or not to share data in their EHRs for research. Understanding how to help patients make informed decisions is particularly important in populations who would be vulnerable to significant risk because of the nature of the data contained in their EHR, or for those who are experiencing a medical condition that may be associated with stigma, such as infertility. A systematic review of nine studies, conducted since 1998, examining willingness to consent to provide access to medical records found wide variation in willingness, with older adults and males being most likely to consent, and people with sensitive medical concerns being least likely to consent [12]. Most of the studies in the systematic review conducted in the United States were published before widespread use of EHRs [12].

In the Belmont Report, the National Commission for the Protection of Human Subjects of Biomedical and Behavioral Research describes three basic ethical principles for research involving human subjects: respect for persons, beneficence, and justice [13]. The Belmont Report further emphasizes the importance of informed consent (IC), assessment of risks and benefits, and selection of participants. IC provides individuals with the ability to exercise their right to be informed, address concerns, and make an autonomous decision about how, when, and at what point in time they will participate in research, if ever. Assessment of risks and benefits by participants can help them decide whether or not to participate in the proposed research. Federal regulations in the United States specify 12 basic elements of IC that must be disclosed to research participants (Box 1), but also indicate that institutional review boards (IRBs) have the latitude to approve consent procedures that alter or waive some or all elements of consent [14]. Several approaches to consent and models of IC documents have emerged and are currently used in research.

With opt-in approaches to consent, there are significant variations in the type and structure of IC documents. The broad consent approach seeks participant consent to a wide variety of uses and assumes consent will cover all uses within biomedical research, including unknown future research. One important consideration for broad consent is the scope. In broad consent, the information within the consent is filled with substantial ambiguity, and the participant may be unaware of the specific uses of their data, and/or for how long their data may be utilized. In contrast, more narrow models of consent involve explaining specific studies in which a participant is asked to partake. A narrow model of consent discusses a study’s potential risks and benefits, the right to withdraw, and additional participant concerns. With this model of consent, participants must be contacted before each additional study on an ongoing basis, which may pose concerns about cost and logistics [15]. Another method is to provide a “menu” of options, such that individuals may preselect the types of research for which they would allow their information and samples to be used (categorical consent), or they may choose to be contacted before their data are used [16, 17]. Furthermore, IC forms may have varying degrees of ‘simplicity.’ Compared to standard IC forms that provide extensive information, simplified consent forms (also known as ‘enhanced’ IC forms) may use: shorter, simple language; larger font; more blank space; active voice; and may place important information at the beginning of the IC and include illustrations to augment the written content [18–20].

Research suggests study participants may frequently not understand information disclosed in the IC process [21–26]. IC form length may be one important factor in participant comprehension. A recent meta-analysis [27] found simplified IC forms were associated with significantly increased understanding compared to standard IC forms. However, other studies have found no differences in comprehension between standard and simplified IC forms [19, 28, 29]. Although it is not clear whether simplified IC forms are associated with comprehension, potential research participants may prefer simplified IC forms because they are easier to read [19]. A recent review found three out of 12 interventions to enhance IC (two multimedia, one simplified IC form) were associated with improved accrual to studies compared to control, while the other nine studies found no effect [29].

Some studies indicate that broad IC forms are preferred to menu IC forms when people are asked to participate in biobanking or genomic research [15, 16]. One study found 41% of people preferred broad consent compared to 29% who preferred study-specific consent, and 25% who preferred menu consent [15]. The most cited reasons for preferring broad IC forms were helping others and convenience [15]. A nationally representative study of US adults found that participants marginally preferred broad IC forms (46%) over study-by-study consent models (44%) and menu IC forms (10%) [16]. Older males were more likely to choose broad IC forms over narrow IC forms. In addition, individuals who chose broad IC forms were more likely to endorse reasons related to altruism and convenience. In contrast, individuals were more likely to opt for study-specific IC forms if they endorsed having concerns about privacy and possible harms from data sharing. This study did not explicitly examine attitudes among individuals with specific health conditions or with sensitive medical information in their EHRs.

More research is needed on the type of consent that is optimal for EHR sharing in populations with sensitive information in their EHR. As vast amounts of EHR data accumulate, research questions that we cannot envision today will be asked in the future. As such, seeking IC for future studies in the era of the EHR will be, to some extent, inherently ambiguous. The challenge will be to determine the optimal IC process that will respect and satisfy the ethical principles and applications articulated in the Belmont Report. Given the breadth of personal, family and genetic information collected as part of the care of couples with infertility, studying the IC preferences of this population may provide invaluable insights into EHR sharing. To date, no studies have been conducted that specifically focus on the preferences of people seeking fertility care with sensitive data contained in their EHRs. There is some indication that study participants may prefer broad and simplified ICs [15, 16, 19], although it is difficult to draw on this research because it was conducted in the context of clinical trials or biospecimen research rather than EHR data sharing.
